# Elastolytic activity of cysteine cathepsins K, S, and V promotes vascular calcification

**DOI:** 10.1038/s41598-019-45918-1

**Published:** 2019-07-04

**Authors:** Pierre-Marie Andrault, Preety Panwar, Neil C. W. Mackenzie, Dieter Brömme

**Affiliations:** 1 Department of Oral Biological and Medical Sciences, Faculty of Dentistry, University of British Columbia, Vancouver, BC V6T1Z3 Canada; 2 Centre for Blood Research, University of British Columbia, Vancouver, BC V6T 1Z3 Canada; 3 Department of Biochemistry and Molecular Biology, Faculty of Medicine, University of British Columbia, Vancouver, BC V6T1Z3 Canada

**Keywords:** Proteolysis, Calcification

## Abstract

Elastin plays an important role in maintaining blood vessel integrity. Proteolytic degradation of elastin in the vascular system promotes the development of atherosclerosis, including blood vessel calcification. Cysteine cathepsins have been implicated in this process, however, their role in disease progression and associated complications remains unclear. Here, we showed that the degradation of vascular elastin by cathepsins (Cat) K, S, and V directly stimulates the mineralization of elastin and that mineralized insoluble elastin fibers were ~25–30% more resistant to CatK, S, and V degradation when compared to native elastin. Energy dispersive X-ray spectroscopy investigations showed that insoluble elastin predigested by CatK, S, or V displayed an elemental percentage in calcium and phosphate up to 8-fold higher when compared to non-digested elastin. Cathepsin-generated elastin peptides increased the calcification of MOVAS-1 cells acting through the ERK1/2 pathway by 34–36%. We made similar observations when cathepsin-generated elastin peptides were added to *ex vivo* mouse aorta rings. Altogether, our data suggest that CatK-, S-, and V-mediated elastolysis directly accelerates the mineralization of the vascular matrix by the generation of nucleation points in the elastin matrix and indirectly by elastin-derived peptides stimulating the calcification by vascular smooth muscle cells. Both processes inversely protect against further extracellular matrix degradation.

## Introduction

Vascular calcification is recognized as an important risk factor that significantly increases the mortality rate by a factor of 3 to 4 in atherosclerosis and up to 60-fold when associated with chronic kidney diseases (CKD) or type II diabetes mellitus^1^. The accumulation of vascular calcium deposits affects arterial elasticity, leading to an increased risk of cardiac failure^2^. Moreover, plaque-associated mineralization is a well-known marker of cardiovascular pathology^3^. It is characterized by the progressive calcification of the walls of elastic muscular blood vessels, such as aorta, carotid, and coronary arteries, resulting in a weakening of vasomotor ability. The calcification process can be classified into two major categories: (i) intimal calcification that is associated with structural changes in the vasculature wall and the proteolytic degradation of extracellular matrix (ECM) proteins like elastin and collagens, and (ii) medial calcification (also known as Mockënberg’s sclerosis), which does not involve ECM remodeling^4^. Intimal calcification is often associated with atherosclerosis and calcified areas are mainly localized in atherosclerotic lesions^5^. Previous work showed that vascular smooth muscle cells (VSMCs), macrophages, and multinucleated cells participate in atheroma plaque formation and progression and are also involved in the regulation of vascular mineralization^6,7^. These plaque-forming cells contribute to the progression of the pathology through the secretion of elastolytic proteases, including matrix metalloproteinases (MMP-2, -9 and -12) and cysteine cathepsins (CatK, S, and V)^8–10^. Human cysteine cathepsins are a group of endolysosomal proteases comprising 11 members (CatB, C, F, H, K, L, O, S, V, W, and X) with various intracellular house-keeping functions, but if secreted are also involved in the cleavage of ECM proteins, such as type I, II, and IV collagens, elastin, fibronectin, and nidogen^11^. CatK, S, and V are among the most potent mammalian elastases and previous work has demonstrated that they represent about 60% of the total elastase activity in plaque-associated VSMCs and macrophages^12^. These cathepsins contribute to the progression of atherosclerosis and to the outcome of several associated complications, such as medial calcification, valvular calcific aortic stenosis, and plaque rupture^10,13–15^. CatK and S have been positively correlated with the development of vascular calcification in the context of CKD^16,17^. However, the precise mechanism by which cysteine cathepsins promote vascular calcification remains elusive.

The aim of the present study is to shed light on the role of elastolytic cysteine cathepsins in vascular calcification. We demonstrated that the degradation of elastin by CatK, S, and V and the resulting disorganization of the elastic fiber promotes its calcification, but also makes the mineralized elastin more resilient to further degradation. In addition, soluble elastin peptides released after digestion of elastin by CatK, S, and V have a stimulatory effect on the calcification of VSMCs.

## Results

### Degradation of mineralized elastin

**Figure 1:**
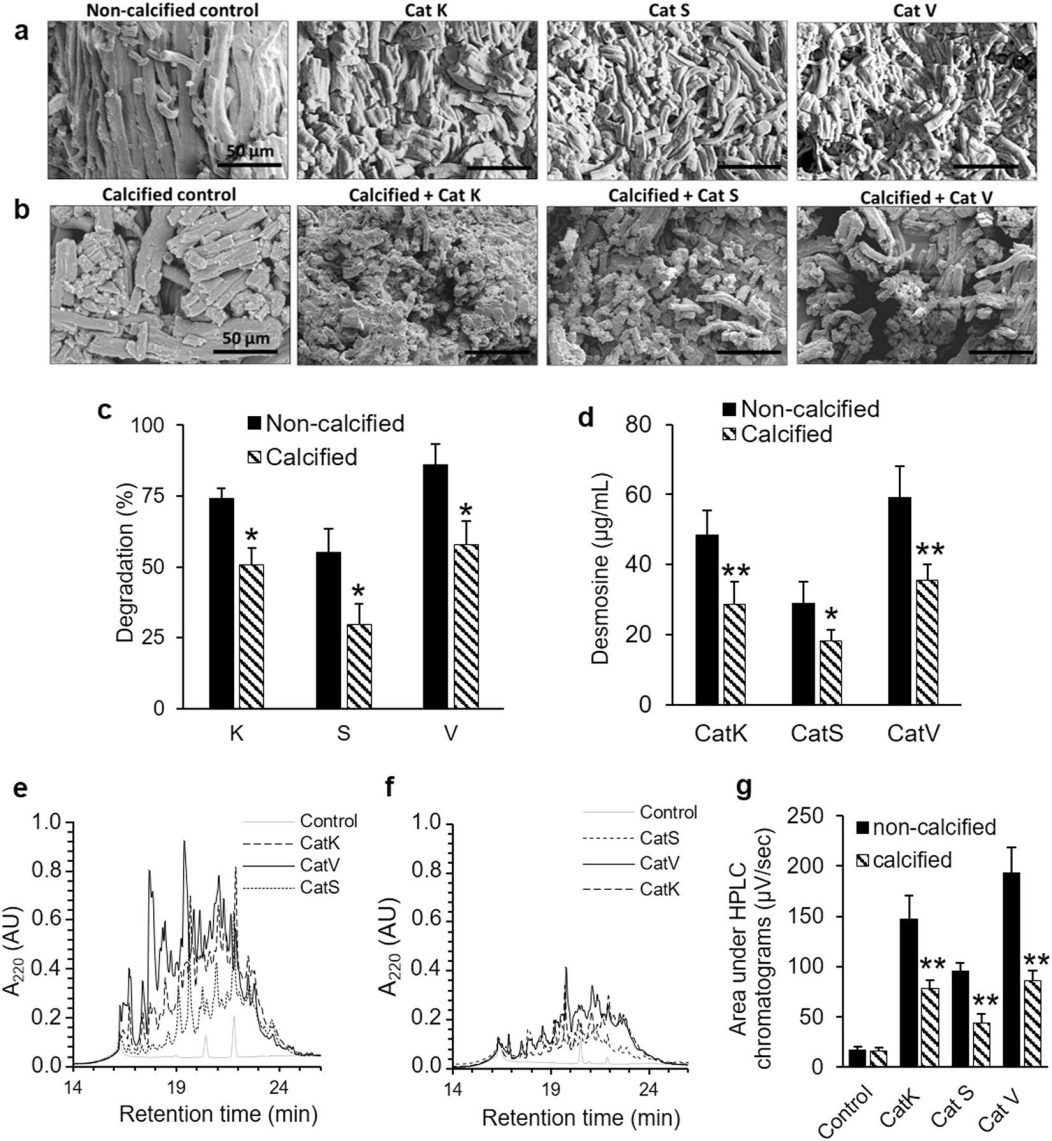
Digestion of non-calcified and calcified elastin by CatK, S, and V. Native or calcified bovine neck elastin (1 mg) was incubated with 2 µM CatK, S, or V in 100 mM sodium acetate pH 5.5, 2.5 mM DTT, 2.5 mM EDTA for 18 h at 37 °C. (**a**) Non-calcified and (**b**) calcified elastin were analyzed by SEM after digestion. (**c**) Remaining elastin weight was measured after digestion and expressed as a percentage of the control. (**d**) Desmosine release in the digestion supernatant was quantified by ELISA. Digestion supernatants from (**e**) non-calcified and (**f**) calcified elastin were loaded on a C-18 column and eluted by HPLC. (**g**) The area under the chromatograms were quantified with Empower software (Waters). Data from non-calcified elastin (n = 6) were compared to the corresponding calcified samples (n = 6) by one-way Mann-Whitney U test. (*p < 0.025, **p < 0.01).

The digestion of elastin by CatK, S, and V considerably changed the structural organization of the elastin fibers as seen on the electron scanning microscopy images (Fig. 1a). All three cathepsins were able to cut the fibers into smaller pieces. Digested fibers presented a drastic reduction in their length and thickness, especially after digestion with CatV, the most potent elastase among human cathepsins. The appearance of calcified elastin was not much different from non-calcified elastin (Fig. 1b). CatK, S, and V were able to degrade the mineralized fibers, however, the mineralization resulted in a protective effect on their further degradation. Degradation fragments derived from mineralized fibers were longer and thicker when compared to native fibers which suggests that they were less degraded. Thus, the mineralization of elastin might constitute a protective mechanism against further proteolytic degradation. Mineralized elastin revealed less degradation (~25–30%) after being subjected to CatK, S, and V when compared to non-calcified elastin (*p* < 0.025) based on the respective remaining weights of the digested and undigested samples (Fig. 1c). In support of this finding, desmosine release from degraded mineralized elastin was also decreased by ~50% when compared to native elastin (*p* < 0.01 for CatK and V, *p* < 0.025 for CatS) (Fig. 1d).

HPLC analysis of CatK- and V-generated hydrolysis products from non-calcified elastin displayed retention times between 16 and 23 minutes, while CatS-generated major fragments were eluted between 19 and 23 minutes (Fig. 1e). The HPLC profiles of the hydrolysis products obtained after digestion of calcified elastin showed a lower overall intensity compared to the native elastin digestion profiles, with less distinguishable major peaks (Fig. 1f). The quantification of the area under the chromatograms showed that the amounts of soluble fragments released from mineralized elastin fibers was reduced by 47% for CatK, 54% for CatS, and 56% for CatV when compared to native elastin (*p* < 0.01) (Fig. 1g).

### Calcification of digested elastin

**Figure 2:**
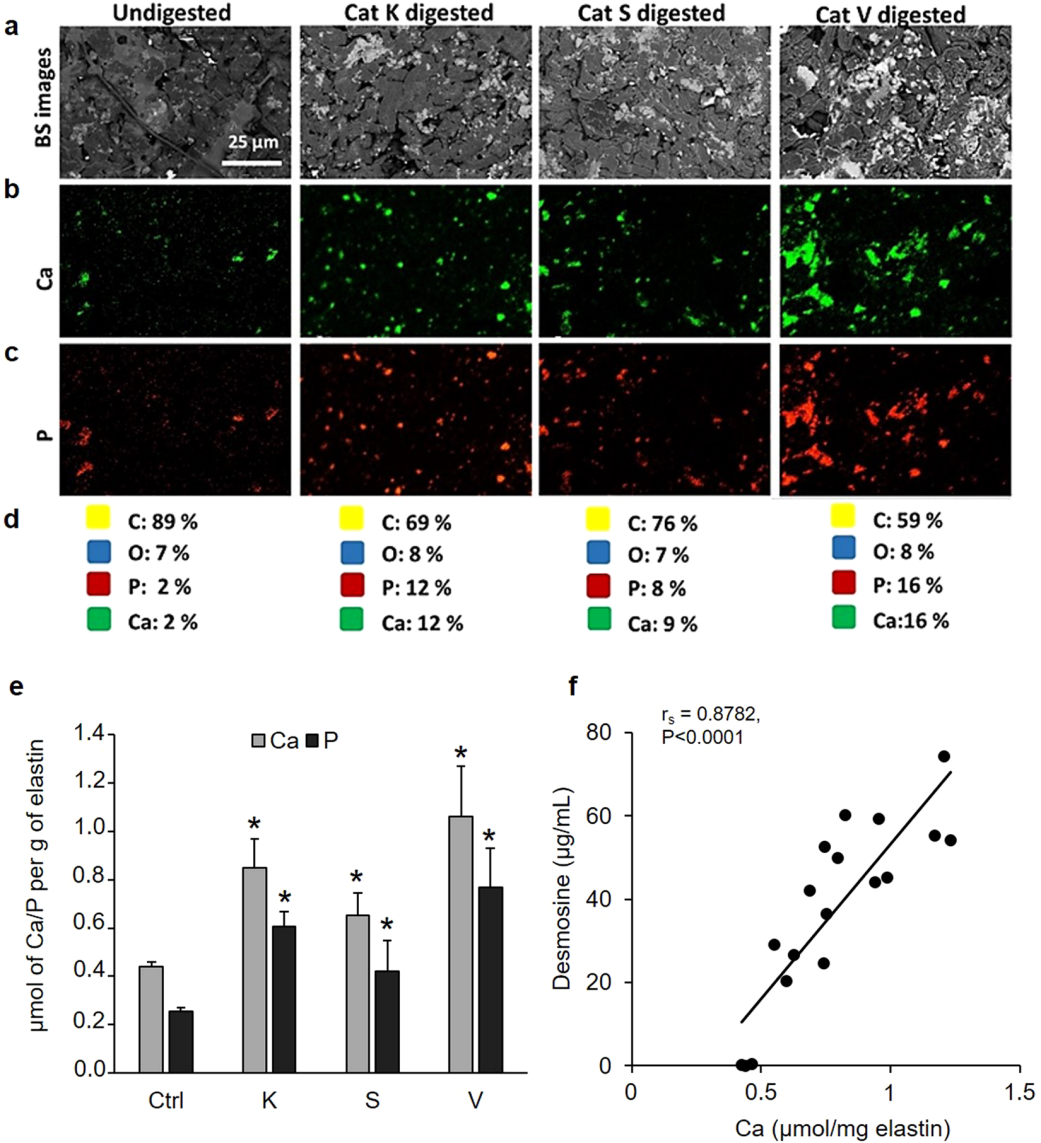
Mineralization of CatK-, S-, and V-digested elastin fibers. Bovine neck elastin was digested by CatK, S, and V prior to mineralization. (**a**) Samples were analyzed by Back Scattered (BS) SEM. EDS analysis confirmed the mineral deposits on calcified elastin fibers highlighting (**b**) calcium, (**c**) phosphate and (**d**) the elemental content was expressed as percentage. (**e**) Intact and digested mineralized elastin was decalcified with 0.6 N HCl and acid extracts were analyzed for calcium and phosphate content (µmol/g of elastin). Pairwise comparison of Calcium/Phosphate levels between control elastin (n = 5) and digested samples (n = 5 for each set) was carried out by Mann-Whitney U test (*p < 0.01). (**f**) The amount of desmosine detected in the digestion supernatant was correlated with the amount of calcium determined in the corresponding sample. The correlation between the two variables was analyzed by the Spearman method.

Our SEM investigations showed that degraded elastin is fragmented into shorter pieces, therefore, we hypothesized that this would increase the number of accessible hydroxyapatite nucleation sites, thus favoring the mineralization process. Back-scattered electron micrographs confirmed that the digestion of bovine neck elastin by CatK, S, and V dramatically increased the amount of mineral deposits (Fig. 2a). We used purified bovine neck elastin as due to its purification procedure it is mostly free of other elastin-associated components such as fibrillins, fibulin, and various glycoproteins that may exert an additional effect on the mineralization. The energy dispersive X-ray spectroscopy (EDS) mapping showed that the mineral nodules were composed of calcium, phosphate, and oxygen, and that these three elements co-localized with each other (Fig. 2b,c). Elastin digested with CatK, S, and V increased both calcium and phosphate elemental content by 10%, 6%, and 14%, respectively (Fig. 2d). EDS results were supported by direct quantification of the calcium and phosphate content of mineralized digested samples (about 1.5 - to 2.4-fold increase for calcium and 1.7 - to 3-fold increase for phosphate; *p* < 0.01) (Fig. 2e). To demonstrate that the addition of the protease protein did not affect the calcification, we incubated the elastin fibers with CatK in the presence of the cysteine protease inhibitor E64. Here, the incubation with L-3-carboxy-*trans*-2,3-epoxypropionyl-leucylamido-(4-guanidino)butane (E64) -inactivated CatK gave a comparable amount of calcification as elastin incubated alone (Fig. S1). As expected, the calcium and phosphate levels measured in digested elastin samples were higher than the control elastin samples (*p* < 0.01). We found a positive correlation between the amount of released desmosine and the degree of elastin calcification (Spearman r: 0.8782, *p* < 0.0001), suggesting that the amount of mineral deposition is directly proportional to elastin degradation (Fig. 2f).

### Characterization of the mineral phase

**Figure 3:**
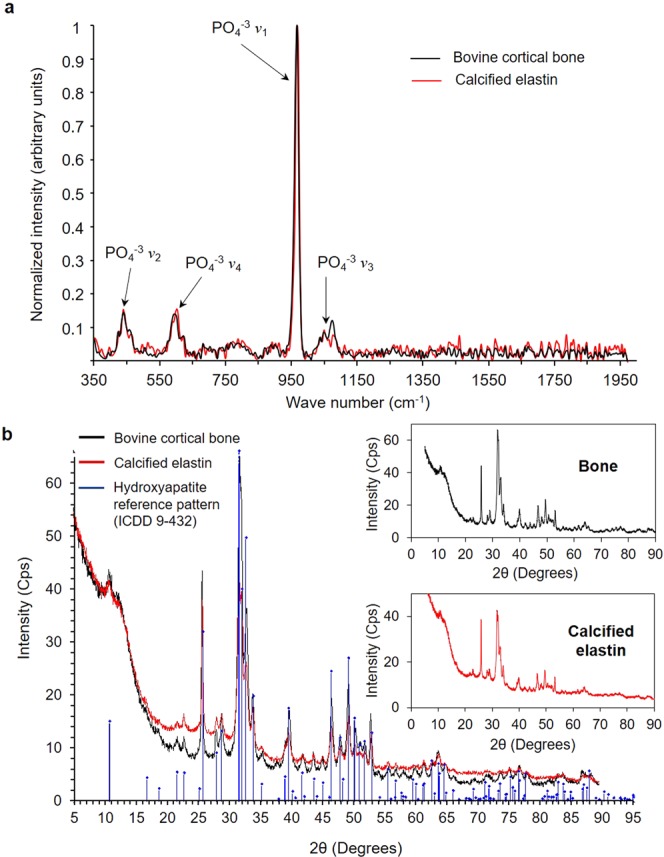
Characterization of the mineral phase of calcified elastin. (**a**) Raman spectra obtained from cortical bone sample (black) and calcified elastin (red) mineral fractions. The peaks corresponding the phosphate anion vibrational frequencies are indicated with arrows. (**b**) Powder X-ray diffraction patterns of cortical bone (black) and calcified elastin (red) mineral fractions. The diffractions patterns were plotted with the hydroxyapatite reference pattern (ICDD 9-432) obtained from PDF-2 2012 database.

To get further insight into the nature of the calcium crystals, we performed Raman spectroscopy analysis on the mineral extracted from calcified elastin. The spectrum obtained from the mineral fraction of calcified elastin is in good agreement with the spectrum obtained from the mineral fraction of bovine bone (Fig. 3a). We observed the characteristic bands corresponding to the phosphate anion vibrational frequencies (*ν*_1_, *ν*_2_, *ν*_3_, and *ν*_4_), which are associated with the different stretching and bending modes of the phosphate-oxygen bonds (Table 1). These measurements are in accordance with previously published hydroxyapatite spectra^18,19^. To strengthen these observations, we performed powder X-ray diffraction on our samples which is a more sensitive method than Raman spectroscopy. The X-ray diffraction spectrum of the mineral phase of calcified elastin was identical to the spectrum obtained from a bone specimen (Fig. 3b). Moreover, both spectra were completely aligned with the reference diffraction pattern of hydroxyapatite (ICDD 9-432). Altogether, these results showed that the mineral phase of calcified elastin is hydroxyapatite and thus highly comparable to the bone mineral.

**Table 1:** Band assignment of Raman spectra from bovine bone and calcified elastin mineral fractions.

Wave number (cm^−1^)	Band assignment
430–465	PO_4_^−3^ *ν*_2_, O-P-O doubly degenerate bending modes
570–627	PO_4_^−3^ *ν*_4_, O-P-O triply degenerate bending modes
968	PO_4_^−3^ *ν*_1_, symmetric P-O stretching
1020–1070	PO_4_^−3^ *ν*_3_, asymmetric P-O stretching

### Calcification of vascular smooth muscle cells

**Figure 4:**
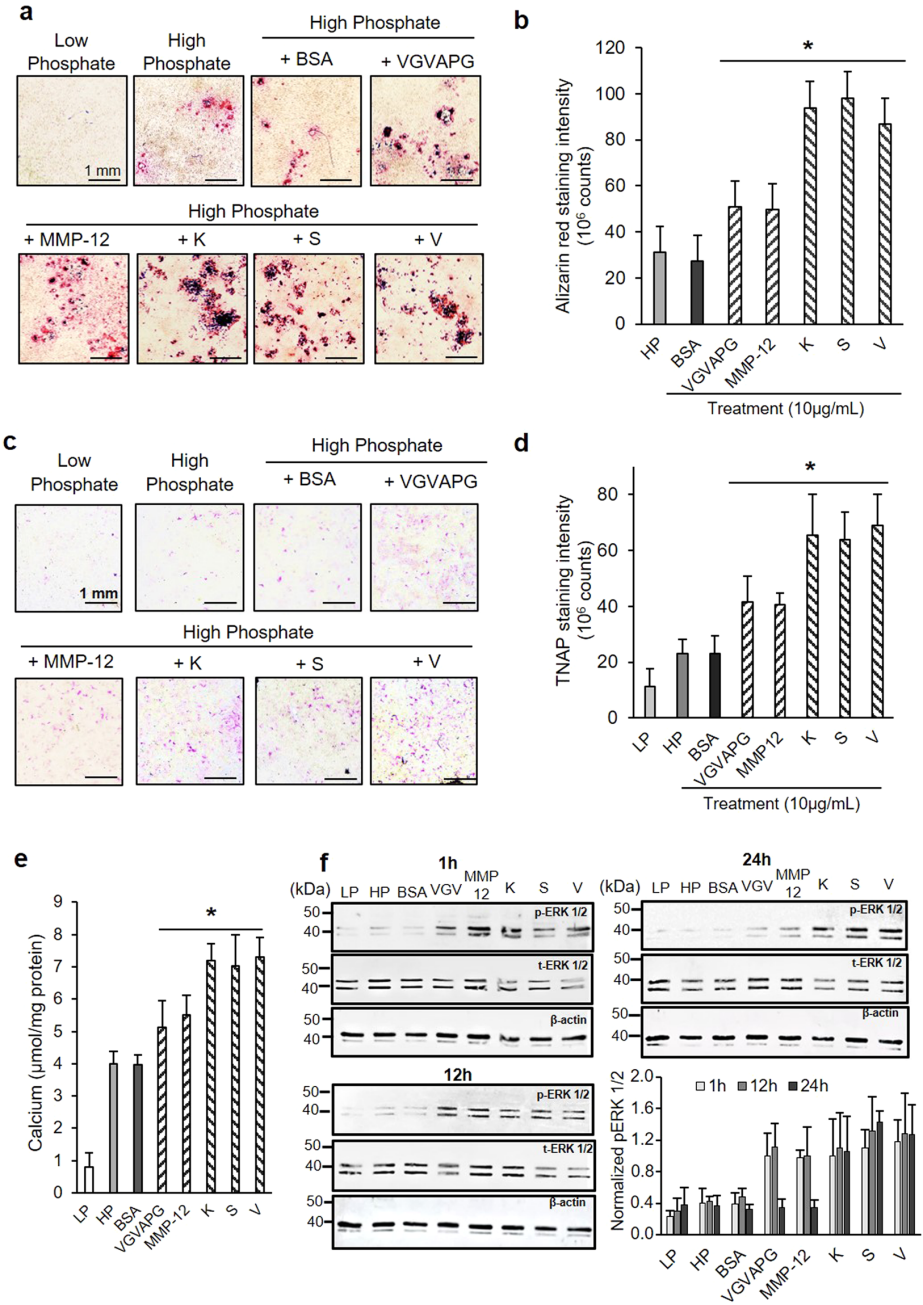
Effects of cathepsin-generated elastin peptides on vascular smooth muscle cell calcification. MOVAS-1 cells were incubated in low phosphate (LP) complete DMEM (1 mM Pi) or high phosphate (HP) complete DMEM (2 mM Pi) in the presence or absence of a tryptic BSA digest, synthetic VGVAPG peptide or CatK-, S-, V or MMP-12-digested elastin (each 10 µg/mL) for 21 days. (**a**) Cell layers were stained with 2% Alizarin-Red pH 4.2 (calcium staining), images were recorded at 20x magnification and (**b**) the intensity of Alizarin-Red-positive staining was quantified with NIS-Elements software (Nikon), data are represented as mean ± SD. (**c**) Cytochemical staining of active alkaline phosphatase was carried out with an alkaline phosphatase detection kit (Sigma-Aldrich) and (**d**) staining intensity was quantified with NIS-Elements software (Nikon), data are represented as mean ± SD. (**e**) Cell layers were decalcified with 0.6 N HCl for 24 h and solubilized calcium was quantified by cresolphthalein assay (Randox) and normalized to protein concentration. (**f**) Lysates from 1 h, 12 h and 24 h-old cell culture were loaded (30 µg total protein) and separated onto 12% acrylamide gel, transferred onto a nitrocellulose membrane and probed with anti-phospho-ERK1/2 (p-ERK1/2) Membranes were then stripped and probed with anti-ERK1/2 antibodies, then stripped again and probed with anti-β-actin antibody (all three panels are showing the same membrane). The intensity of the immunoreactive bands was quantified by ImageJ software (NIH software) and normalized to the β-actin corresponding signal. Comparison between control HP (n = 4) and peptides treated HP samples (n = 4 for each set) was carried out with the one-way Mann-Whitney U test (n.s.: non-significant, *p < 0.05).

We hypothesized that the degradation of vascular elastin by cathepsins generates bioactive elastin fragments also known as elastokines. Previous reports showed that bovine neck elastin hydrolysate can stimulate the mineralization of rat aortic smooth muscle cells and fibroblasts^20,21^. Therefore, we digested bovine neck elastin fibers with CatK, S, and V and then used the soluble elastin fragments to stimulate VSMCs. The same experiment was repeated with MMP-12 to compare the contribution of cathepsins and the major elastolytic matrix metalloproteinase in elastokine-dependent cellular-regulated mineralization. As a negative control, we used a tryptic digest of BSA to assess any potential effects solely due to the introduction of foreign peptides. We exploited the MOVAS-1 cell line that has previously been described as a suitable VSMC calcification model^22^. Cells cultivated under high phosphate (HP, pathological) conditions showed the presence of Alizarin-Red positive calcium nodules, while no staining was observed in cells cultivated under low phosphate (LP, physiological) conditions (Fig. 4a). Moreover, cells cultivated in low phosphate containing medium did not show any detectable calcium deposits after stimulation with 10 µg/mL BSA peptides, VGVAPG peptide or CatK-, S-, V- and MMP-12-generated elastin peptides (Fig. S2). MOVAS-1 cells cultivated in high phosphate medium and stimulated with 10 µg/mL of elastin digests showed an increased amount of Alizarin-Red positive calcium nodules on their surface compared to the non-stimulated control but no stimulating effect was observed when cells were treated with peptides originating from BSA (Fig. 4a,b). In more detail, we observed a ~2-fold increase upon MOVAS-1 cells mineralization when cells were stimulated with the synthetic elastokine VGVAPG and elastin peptides generated by MMP-12 (*p* < 0.05; Fig. 4a,b). The stimulating effect was even higher when cells were incubated with elastin peptides generated by the elastolytic cathepsins (~2.8- to 3.2-fold increase, *p* < 0.05). Incubation of MOVAS-1 cells under high phosphate conditions also increased the expression of tissue non-specific alkaline phosphatase (TNAP) when compared to low phosphate conditions, as demonstrated by cytochemical staining of active forms of TNAP (Fig. 4c). When compared to the untreated control, the amount of TNAP signal increased about 1.8-fold after stimulation with elastin peptides generated by MMP-12 and the synthetic VGVAPG peptide (*p* < 0.05) and about 3-fold with the cathepsin-generated elastin fragments (*p* < 0.05) (Fig. 4d). A similar trend of changes was observed for the calcium deposition (measured as acid solubilized calcium from the cell layer). The amount of acid solubilized calcium from cell layers increased by ~28–34% in cells stimulated with the MMP-12-generated elastin digest and the VGVAPG peptide and by ~60–65% in cells stimulated by cathepsin-produced elastin peptides. No effect was observed with the BSA digest (Fig. 4e).

Previous studies showed that the mineralization of VSMCs was regulated by the extracellular signal-regulated kinase 1/2 (ERK1/2) pathway^23–25^. We detected a higher level of phosphorylated ERK1/2 (pERK1/2) (~2.5- to 3.5-fold increase) when cells were stimulated with cathepsin/MMP-12-generated elastin peptides and VGVAPG peptide compared to non-stimulated or BSA peptide-treated cells after 1 h and 12 h (Fig. 4f). Interestingly, stimulation of MOVAS-1 with cathepsin-generated elastin peptides prolonged the life-span of pERK 1/2 up to 24 h against 12 h when cells were exposed to the VGVAPG peptide or MMP-12 generated elastin peptides. When the same experiments were performed in the presence of the ERK inhibitor, FR180204, the stimulating effect of all elastokines was disrupted (Fig. S3). This observation supports the fact that cathepsin-generated peptides promote the mineralization of MOVAS-1 cells through the ERK1/2 pathway.

### Mineralization of mouse aortic tissue

**Figure 5:**
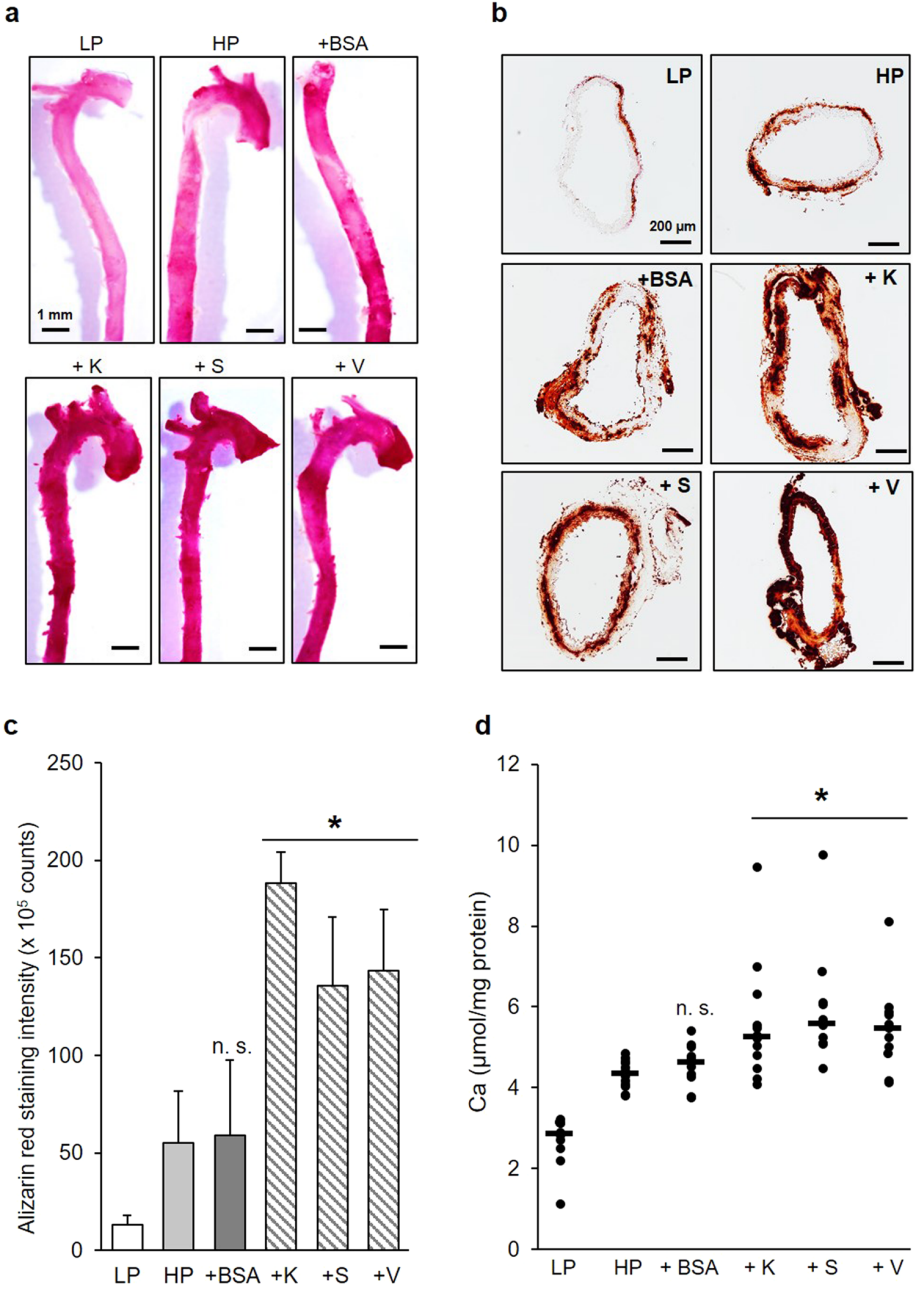
Effect of cathepsin-generated elastin peptides on the calcification of aortic tissue. Aortas from 3-month-old C57BL6 mice were incubated in low phosphate (LP) complete αMEM (1 mM Pi) or in high phosphate (HP) αMEM (2 mM Pi) in the presence or absence of tryptic BSA peptides or CatK-, S-, or V-digested elastin for 12 days at 37 °C. (**a**) Aortas were washed with PBS, fixed with 10% buffered formalin for 24 h and stained with 2% Alizarin-Red pH 4.2 and images were recorded at low magnification. (**b**) 4 mm fragments from descending aortas were embedded in paraffin and cut into 5 µm sections and stained with Alizarin-Red. Images were recorded at 100x magnification. (**c**) Alizarin-Red intensity from 10 sections for each condition was quantified with NIS-Elements software (Nikon); data are represented as mean ± SD. (**d**) 1 mm aorta sections were decalcified with 0.6 N HCl for 24 h and solubilized calcium was quantified by a cresolphthalein assay (Randox) and normalized to protein concentration. Comparison between control HP (n = 10) and peptides treated HP samples (n = 10 for each set) was carried out with the one-way Mann-Whitney U test (n.s.: non-significant, *p < 0.01).

**Figure 6:**
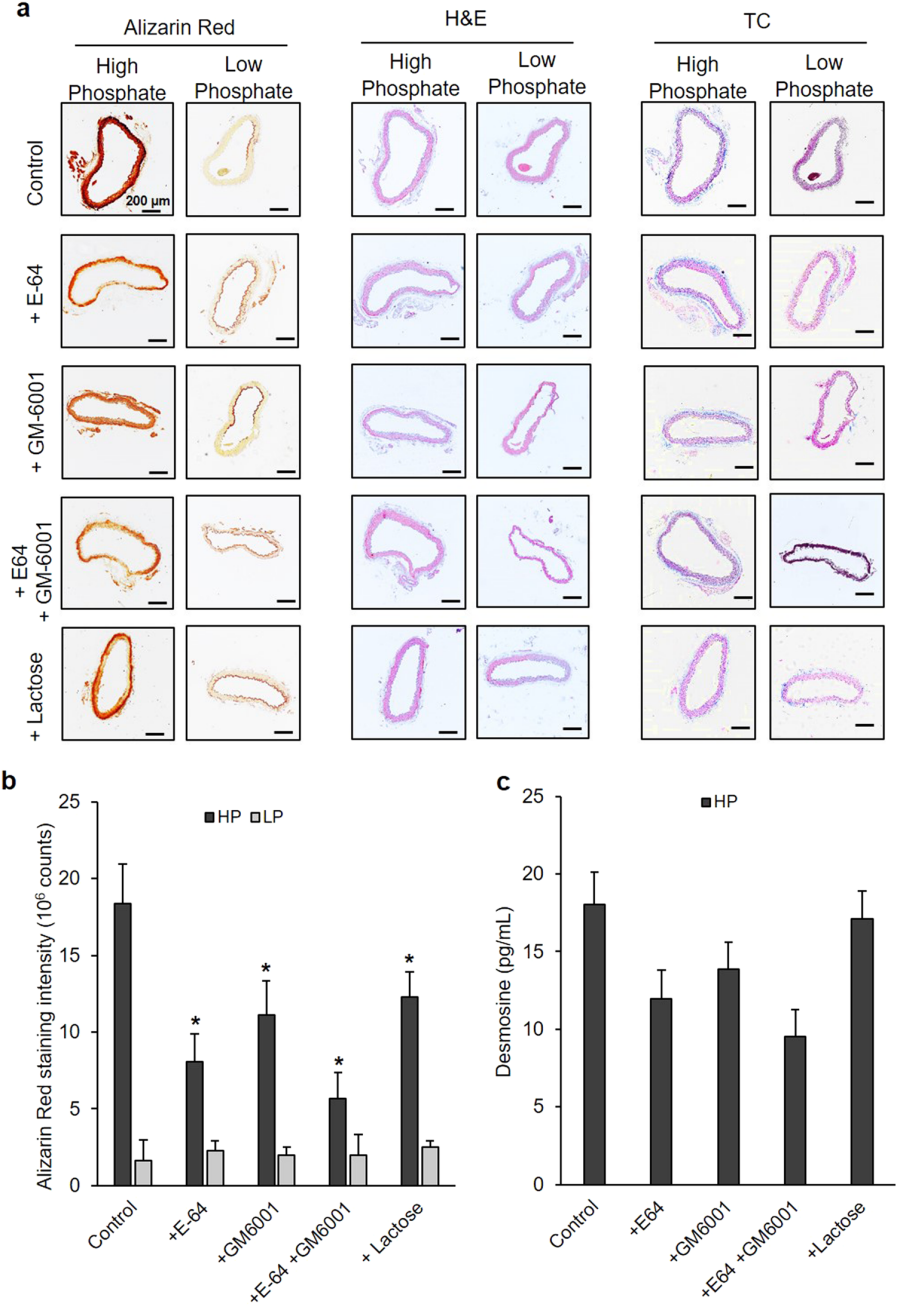
Effects of cysteine cathepsin and MMPs inhibition on aortic ring calcification. 4-mm-long aortic rings were incubated in low phosphate (LP) complete αMEM (1 mM Pi) or in high phosphate (HP) αMEM (2 mM Pi) in the presence or absence of E64 or GM6001 (10 µM) or lactose (50 mM) for 12 days at 37 °C. (**a**) 5 µm sections were stained with hematoxylin and eosin (H&E), Masson’s Trichrome (TC) and Alizarin-Red. Images were recorded at 100x magnification. (**b**) Alizarin-Red intensity was quantified with NIS-Elements software (Nikon), data are represented as mean ± SD. Comparison between control HP (n = 20) and peptides treated HP samples (n = 20 for each set) was carried out with the one-way Mann-Whitney U test (*p < 0.01). (**c**) The amount of desmosine in the culture medium was determined by ELISA.

To assess the potential effects of cathepsin-generated elastokines on vascular tissue mineralization, we used an *ex vivo* model of mouse aorta cultivated under calcifying conditions. Incubation of the whole aorta at high phosphate conditions resulted in Alizarin-Red positive calcium deposits on the wall of the aortic tissue, while incubation at low phosphate conditions showed only minor staining (Fig. 5a). However, when aortas were incubated in the presence of CatK-, S-, and V-generated elastin peptides, Alizarin-Red staining dramatically increased when compared to the non-treated or BSA peptide-treated controls. Investigations on tissue sections stained with Alizarin-Red showed comparable results (Fig. 5b). The calcium deposits were mainly localized in the elastic laminae where VSMCs reside. Calcium deposits were also found in the adventitia below the elastic laminae in the samples stimulated with cathepsin-generated elastin digests. The quantification of Alizarin-Red staining showed that treatment of aortic segments with elastin peptides generated by CatK, S, and V increased the amount of elastic tissue mineralization by factors of ~3.4, ~2.4, and ~2.6, respectively, when compared to non-treated aortic segments (*p* < 0.01) (Fig. 5c). The quantification of tissue-associated calcium in aortic rings showed only minor calcium deposits after incubation in low phosphate medium (median: 2.85 µmol Ca/mg protein) and increased, as expected, in high phosphate conditions (median: 4.35 µmol Ca/mg protein). The degree of calcification of BSA peptide-stimulated aortic rings was not different from the control (median: 4.63 µmol Ca/mg protein). On the other hand, the median values of calcium deposits in the aortic rings were increased by ~20–30% when stimulated with elastin peptides generated by CatK, S, and V (median: 5.25, 5.59 and 5.46 µmol Ca/mg protein respectively) and compared to non-treated controls (*p* < 0.01) (Fig. 5d). When aortic rings under high phosphate conditions were incubated with the cysteine protease inhibitor, E64, the MMP inhibitor, GM6001, or both inhibitors, the amount of medial and intimal calcification was reduced. Quantification of Alizarin-Red staining (Fig. 6a) showed that E64, GM6001 and the mixture of both inhibitors reduced the formation of calcified nodules by ~52%, ~33% and ~67% respectively (*p* < 0.01) (Fig. 6b). When lactose was used to block the effect of the elastin binding receptor (EBR) that transduces the response to elastin peptides signal, tissue calcification was reduced by 34% (*p* < 0.01). Moreover, quantification of the released desmosine showed that E64, GM6001 and a mixture of both inhibitors substantially decreased the amount of elastin degradation by 40%, 35% and 47% respectively compared to the control conditions but was, as expected, not affected by the lactose treatment (Fig. 6c). On the other hand, based on hematoxylin-eosin (HE) and trichrome (TC) staining, we did not observe any morphological changes in the presence of the inhibitors when compared to the control conditions (Fig. 6a). High magnification images (x 1000) showed that calcium deposits are located on the elastic fibers (Fig. S4). Moreover, we did not see any changes in the cellular content suggesting that neither the high phosphate nor the inhibitor treatment had any effect on the viability of the cells. Sirius-Red staining showed that the collagen content was not different between the conditions tested; therefore, the observed differences in calcium levels were not attributed to the collagen content (Fig. S5). We observed some Alizarin-Red positive calcium deposits in the low phosphate control but the inhibitor treatment did not show any effect.

## Discussion

During atherosclerosis, the formation of atheroma plaques is often associated with the degradation of vascular wall constituents. Ourselves and others have shown that cysteine cathepsins are involved in the development of atherosclerotic lesions^12,13,26,27^. Moreover, gene deletion or inhibition of CatK, L, and S in ApoE^−/−^ and LDL receptor^−/−^ mice was associated with less vascular elastin damage and plaque formation^13,14,28,29^. However, no links have been established between the remodeling of vascular tissue by cysteine cathepsins and the subsequent vascular mineralization. The therapeutic strategies currently studied to prevent or regress vascular calcification target the initial pathologic causes such as chronic kidney disease and atherosclerosis^30^. Statins have been investigated in human patients suffering from plaque-associated vascular calcification and have shown limited therapeutic benefits^31,32^. Therefore, it is essential to understand both the molecular and cellular mechanisms associated with plaque formation and its subsequent calcification.

Hyperphosphatemia is linked to arterial stiffness in patients suffering from CKD associated with cardiovascular side effects including arrhythmia, cardiomyopathy, and left ventricle hypertrophy^33,34^. This suggests that a high phosphate level in serum is a relevant risk factor for vascular calcification^35^. We did not change the amount of calcium in our mineralization assay because it is a tightly regulated ionic species in blood plasma. Moreover, serum calcium levels of atherosclerotic patients or patients of other cardiovascular associated pathologies are within the normal range whereas phosphate levels are often increased^36,37^.

Using SEM, we showed that calcium crystal depositions on elastin fibers displayed an uneven pattern suggesting the presence of preferential nucleation sites. It has been proposed that the glycine-rich neutral sequences in tropoelastin constitute a calcium binding site, thus promoting mineralization^38,39^. We used purified elastin to attribute the mineralization-promoting effect of protease-mediated degradation exclusively on elastin without interference of other ECM and elastin-fiber-associated proteins such as fibrillin, fibulin, and other glycoproteins. Our Raman spectroscopy and X-ray diffraction analysis on the mineral phase of calcified elastin showed that it was composed of hydroxyapatite and highly comparable to bone apatite. These observations are in accordance with previous reports that analyzed the composition of calcified elastin *in vitro* and *in vivo*^38,40^. It has been suggested that the high glycine content in collagen and elastin may explain why they both display hydroxyapatite deposits after mineralization^39^. Furthermore, our SEM and EDS investigations clearly showed that the degradation of elastin by CatK, S, and V increases the amount of mineralization. We hypothesized that the proteolytic degradation of elastin leads to the disruption of the elastin structure and thus may increase the overall calcification prone surface as well as the number of accessible nucleation sites (glycine-rich sequences). Conversely, we observed that mineralized elastin was more resilient to cathepsin-mediated degradation. Previous studies using human lung elastin showed that CatK, besides other potent elastases like neutrophil elastase and MMP-12, displayed preferential cleavage sites with a glycine in the P1 position^41^. Unpublished data from our laboratory also show the preferential cleavage of elastin after glycine residues by all three elastolytic cathepsins (Panwar *et al*., manuscript in preparation). Adversely, the mineralization of glycine-rich sites might block further proteolytic cleavage in these particular regions.

The degradation of elastin can generate bioactive fragments called elastokines that contribute to the progression of atherosclerosis, including vascular calcification^42^. Elastin derived peptides have been shown to stimulate the osteogenic response of rat VSMCs and fibroblasts when combined with TGF-β1^20,21^. The generation of elastokines has been previously established for serine-dependent elastases such as human neutrophil elastase, proteinase-3, and cathepsin G as well as metalloproteases (MMP-12)^43,44^, but nothing has been demonstrated yet for cysteine cathepsins. Using an *ex vivo* model of mouse aortic rings, we have shown that the soluble fractions of elastin generated by the digestion of elastin by CatK, S, and V stimulate aortic tissue calcification, suggesting that all three cathepsins can generate soluble, biologically active peptides from insoluble elastin. A similar suggestion was previously made in a model of chronic renal disease using ApoE^−/−^ CatS-deficient or ApoE^−/−^ mice treated with specific CatS inhibitors, where these mice showed a significantly reduced vascular calcification associated with fewer aortic elastin breaks^17,45^.

The mineralization of the vascular tissue mostly involves VSMCs and their phenotypic switch into osteocyte/chondrocyte-like cells^4^. The mineralization of MOVAS-1 cells treated with CatK-, S-, and V-generated elastin peptides was higher when compared to non-treated cells or cells treated with BSA peptides. Previous reports showed that the activation of the extracellular signal-regulated kinase 1/2 (ERK1/2) is a key regulator of osteogenic transition and mineralization of VSMCs^23–25^. In addition, it has been demonstrated that elastin peptides are able to induce ERK1/2 phosphorylation in VSMCs and other cell types such as fibroblasts^46–48^. In this study, we showed that the stimulation of MOVAS-1 cells with cathepsin-generated elastin peptides increased the amount of phosphorylated ERK1/2 (~2.5- to 3.5-fold increase). We also demonstrated that elastin peptides released by cysteine cathepsins could stabilize the phosphorylated form ERK 1/2 for at least 24 h in contrast to only 12 h for the VGVAPG peptide and MMP-12 peptides. This can explain why we observed a higher stimulating effect upon calcification with cathepsin-generated elastin peptides. The observations carried out on MOVAS-1 cells were also corroborated by our *ex vivo* assays on an aortic ring treated with cathepsin-generated elastin peptides. As expected, global inhibition of cysteine cathepsin activity using E64 decreased the overall calcification of the aortic tissue. In comparison, blocking the global activity of MMPs with GM6001 had a lesser inhibiting effect. The blocking effect of GM6001 upon desmosine release was comparable to E64, however, our cellular data showed that elastokines released by MMP-12 have a lesser stimulating effect on VSMCs calcification which may explain why GM6001 had a lesser inhibitory effect on calcification. Cysteine cathepsins can activate pro-MMPs and conversely MMPs can convert cysteine cathepsins zymogens into active forms^49–52^. Thus, inhibiting one type of proteases may have an indirect effect upon the activity of other families of proteases. This may explain why we do not see an additive effect upon desmosine release when both MMP and cysteine protease inhibitors are added together. Moreover, vascular smooth muscle cells and endothelial cells express serine-dependent elastases (human neutrophil elastase, HtrA1)^53–55^. This is probably the reason why the desmosine release was not completely inhibited in the presence of a combination of E64 and GM6001.

We did not see any modification in the architecture of the vascular wall, indicating that limited VSMC-related proteolysis is enough to promote calcification of elastin without displaying marked elastic lamina fragmentation. A similar observation was made in a previous report, where GM6001 was used indicating that also MMPs contribute to the calcification of elastin^56^. Moreover, blocking of the elastin binding receptor using lactose reduced calcification which suggest that the generation of elastokines from vascular elastin degradation directly contributes to the calcification process. We observed a decrease of calcification using both proteases inhibitors and lactose-induced blocking of the EBR which suggest that mineralization was stimulated by both the generation of additional nucleation points and the release of bioactive peptides from elastin.

In conclusion, the present study showed that (i) limited proteolysis of fibrous elastin by CatK, S, and V exposes more nucleation sites for mineralization, (ii) the mineralization of elastin constitutes a partially protective mechanism against further proteolytic degradation, and (iii) CatK, S, and V can generate soluble elastin-derived fragments that stimulate the calcification of vascular smooth muscle cells. Future work will investigate the effect of selective anti-elastolytic inhibitors of CatK, S, and V on plaque-associated calcification in an ApoE^−/−^ mouse model.

## Materials and Methods

### Recombinant cathepsin expression and purification

The detailed protocol of the expression and purification of recombinant enzymes is provided in the supplementary data section.

### Mineralization and digestion of elastin fibers

Congo-Red elastin or bovine neck elastin (Sigma-Aldrich Canada, Oakville, Ontario, Canada) were resuspended either in complete Minimum Essential Medium (MEM) medium (1x MEM, 10% Fetal Bovine Serum, Low phosphate medium) or in complete MEM with the phosphate concentration adjusted to 2 mM (High phosphate medium). Media were supplemented with 0.02% sodium azide to avoid bacterial growth and mineralization was carried out at 37 °C for 6 days. The elastin was then thoroughly washed 3 times 24 h with ultra-pure water to remove any loosely bound precipitated deposits and calcium was extracted with 0.6 N HCl for 24 h at room temperature. Calcium was quantified using a Randox calcium colorimetric assay kit (Randox Laboratories Ltd., Crumlin, UK) and normalized to elastin weight. Incubation of native or calcified bovine neck elastin (20 mg/mL) was carried out at 37 °C for 18 h with CatK, S, and V (2 µM) in 100 mM sodium acetate pH 5.5 containing 2.5 mM DTT and 2.5 mM EDTA (activity buffer). Samples were centrifuged at 13,500 g for 15 minutes and half of the supernatant was loaded onto a C18 Luna^®^ column (Phenomenex, Torrance, California, USA) in water acidified with 0.1% trifluoroacetic acid (TFA). The elution of the hydrolysis products was accomplished with an increasing gradient (0 to 100%) of 90% acetonitrile conditioned with 0.1% TFA in 30 minutes at a constant flowrate of 0.5 mL/min using a Waters Alliance 2695 HPLC system (Waters, Mississauga, Ontario, Canada). The area under the HPLC chromatograms was quantified with Empower software (Waters). The other half of the supernatant was analyzed by desmosine ELISA as previously described^13^. The remaining elastin pellets were dried and weighed to estimate the loss of insoluble material after digestion.

### Structural and chemical analysis of native and calcified elastin fibers

Calcification and structural modifications of elastin fibers were investigated by scanning electron microscopy (SEM) on a Helios NanoLab™ 650 (FEI, Hillsboro, Oregon, USA). Samples were prepared for SEM analysis as previously described^57^, mounted on a metal stub using adhesive carbon tape, and coated with Au/Pd using a Leica EM MED020 coating system (Leica Microsystems Inc. Ontario, Canada). Imaging was carried out using a voltage of 3–5 kV. Calcified surfaces were quantified using ImageJ software (NIH, Bethesda, MD, USA). The elemental composition of calcified elastin was analyzed by Energy Dispersive X-ray spectroscopy (EDS) on the same instrument using the EDAX TEAM™ Pegasus system (EDAX Inc., Mahwah, New Jersey, USA) for analysis. Samples were mounted on a metal stub and coated with carbon using the same method as described above. Maps were collected at 10 kV with a probe current of 0.2 µA at a working distance of 4 mm to identify the location and amount of calcium, phosphorus oxygen, and carbon.

### Characterization of the mineral phase

The characterization of the crystal phase of the mineral deposits was carried out by Raman spectroscopy and powder X-ray diffraction. To avoid excessive background due to the organic character of elastin, calcified elastin was deproteinized by treatment with 0.5 M sodium hydroxide for 24 h at 70 °C. The remaining calcium crystals were washed with ultrapure water until the pH became neutral again. The minerals were then resuspended in 100% ethanol and dried. As a control, the same treatment was realized on powdered bovine cortical bone. Confocal Raman spectra were collected through a fiber coupled Olympus BX-51 microscope with an excitation wavelength at 785 nm. An average of 18 spectra per sample with 5 second exposure times were acquired though a Princeton Instruments Acton SP2300 with a PIXIS-100 CCD camera. Post-acquisition processing included iterative baseline correction and denoising using discrete wavelet transform. The X-ray diffraction pattern from the powdered samples was generated with a Bruker D8 Advance X-ray diffractometer (Bruker Ltd, Milton, Ontario, Canada) powered at 40 kV/40 mA and emitting Kα_1_ & Kα_2_ radiations. The diffraction signal was acquired with a LynxEye silicon strip detector and data were analyzed with Bruker DiffracPlus EVA software (Bruker) working on the International Center for Diffraction Data (ICDD), Powder Diffraction File (PDF)-2 2012 database.

### Mouse aorta rings culture

Descending aortas from 3 months-old C57Bl6 mice were immediately removed after sacrificing the animals. Aortas were washed a minimum of 3 times in phosphate-buffered saline PBS and the remaining fat tissue and fibrous tissue were removed surgically. Clean aortas were either left intact or cut into 1- to 4-mm-long rings and transferred in complete αMEM medium (1X αMEM, 10% fetal bovine serum (FBS), 1% streptomycin/penicillin) supplemented or not with phosphate (final concentration: 2 mM) and 7 U/mL calf intestinal alkaline phosphatase (Promega, Madison, Wisconsin, USA) in the presence or absence of the pan cathepsin inhibitor, L-3-carboxy-*trans*-2,3-epoxypropionyl-leucylamido-(4-guanidino)butane (E64, Sigma-Aldrich). Cultivation under high phosphate conditions was carried out in the presence or absence of bovine serum albumin (BSA) tryptic digest or elastin cleavage products of CatK, S, or V (10 µg/mL). Inhibition of endogenous cysteine cathepsin and MMP activity was carried out in the presence of 10 µM E64 and 10 µM GM6001 respectively. Blockade of the Elastin binding receptor (EBR) was performed in the presence of 50 mM lactose. Both, inhibitors and peptides, were added fresh during each media change to maintain constant assay conditions (reflecting the continuous elastokine release and its blocking by inhibitor treatment under *in vivo* conditions). Aortic tissues were cultured for 12 days with the medium changed every 2–3 days. Aorta were then thoroughly washed with PBS and calcium deposits were extracted with 0.6 N HCl and quantified as described in previous sections and normalized to protein concentration. Uncut aortas were fixed in 4% formaldehyde for 1 h at 4 °C and stained with 2% Alizarin-Red pH 4.2 (calcium staining). Unbound dye was washed out with water. Aorta segments (4-mm-long) were embedded in paraffin, cut into 5 µm thick sections and mounted on glass slides (Superfrost/Plus, Fisher Scientific, Ottawa, Ontario, Canada). Sections were stained with either hematoxylin and eosin (H&E), Masson’s Trichrome (TC), Sirius-Red (collagen staining), or 2% Alizarin-Red pH 4.2. Images were recorded with a Nikon Eclipse Ci microscope equipped with a DS-Ri2 camera (Nikon, Mississauga, Ontario, Canada) under 100x magnification and analyzed with NIS-Elements software (Nikon).

### Vascular smooth muscle cells MOVAS-1 culture

MOVAS-1 cells (1.10^4^ cells/cm²) were grown in a 24-well plate on 13 mm diameter glass coverslips in complete DMEM media containing 10% FCS, 1% streptomycin/penicillin supplemented or not with phosphate (final concentration: 2 mM), and 7 U/mL calf intestinal alkaline phosphatase (Promega, Madison, Wisconsin, USA). Cells were grown for 21 days in the presence or absence of a synthetic VGVAPG peptide (Genscript, Piscataway, New Jersey, USA) bovine serum albumin (BSA) tryptic digest or elastin cleavage products of CatK, S, V (10 µg/mL). MMP-12 digest was obtained after incubation of bovine neck elastin (20 mg/mL) with activated MMP-12 (R&D system, Oakville, Ontario, Canada) (1 µM) at 37 °C for 36 h and used as described previously. Both inhibitor and elastin digests were added fresh during each media change for the same reason stated in the previous section. The same experiments were carried out in the presence of 1 µM ERK inhibitor FR180204 (Sigma-Aldrich). The pH of the medium was controlled regularly and never exceeded 7.4–7.5 by opposition to the statement made in one former study^58^. Cell layers were washed with PBS and decalcified with 0.6 N HCl for 24 h prior to calcium quantification. Cells were washed twice with PBS to remove medium, fixed with 4% paraformaldehyde, and stained with 2% Alizarin-Red pH 4.2. The cytochemical staining of active TNAP was carried out with an alkaline phosphatase detection kit (SCR004, Sigma-Aldrich) following the manufacturer’s instructions. All images were recorded and analyzed as mentioned in previous sections.

### Western blot analysis

MOVAS-1 cell lysates from 1 h, 12 h, and 24 h old cultures (30 µg of protein) were separated on a 12% polyacrylamide gel under reducing conditions and transferred onto a nitrocellulose membrane (Hybond-ECL, Amersham, Mississauga, Ontario, Canada). The membrane was blocked with 5% non-fat milk in PBS and probed with either rabbit anti-phospho-ERK1/2 (1:1000, Abcam), rabbit anti-ERK1/2 (1:1000, Abcam), or rabbit anti-β actin (1:5000, ThermoFisher, Burlington, Ontario, Canada) antibodies in PBS, 5% non-fat milk, and 0.1% Tween 20 overnight at 4 °C. Anti-rabbit IgG-HRP (Abcam) was used as a secondary antibody and membranes were incubated for 1 h at room temperature. Finally, membranes were developed by chemiluminescence (Amersham).

### Statistical analysis

Pairwise comparison with the control condition was carried out by one-way Mann-Whitney U test. A *p* value < 0.05 was considered significant. Correlation coefficient was determined by Spearman correlation analysis. Sample sizes are stated in the figure legends.

### Ethical approval

All animal experiments were conducted in accordance with the ethical guidelines for animals of the Canadian Council on Animal Care (CCAC) and approved by the University of British Columbia.

## Supplementary information

Supplementary data

## Data Availability

The authors declare that all the data, material and methods of the present study are available in this manuscript.
