# Supplementary material for: Elastolytic activity of cysteine cathepsins K, S, and V promotes vascular calcification
Source: Sci Rep. 2019 Jul 4;9:9682. doi: 10.1038/s41598-019-45918-1 (PMC6609650; doi:10.1038/s41598-019-45918-1)

Elastolytic activity of cysteine cathepsins K, S, and V promotes vascular calcification

Pierre-Marie Andrault, Preety Panwar, Neil C. W. Mackenzie and Dieter Brömme

Supplementary information

Supplementary material

Recombinant cathepsin expression and purification

Recombinant CatK and V were cloned into the pPic9K vector and expressed in *P. pastoris* as previously described[1,2]. The CatS pRSET B expression vector was kindly provided by Dr. Klaus Shilling (Institut für Biochemie, Klinikum der Friedrich-Schiller-Universtität, Jena, Germany). Human recombinant CatS was expressed as inclusion bodies in *E. coli* strain BL21(DE3)pLysS following the auto-induction method described by F. W. Studier[3]. Briefly, the CatS expressing strain was grown for 24 h at 37°C in minimal P-0.5G medium (1 mM $MgSO_4$, 50 mM Na_2HPO_4, 50 mM KH_2PO_4, 25 mM $(NH_4)_2SO_4$, 0.5% glucose, 0.1x trace metal mix, 100 µg/mL methionine, 35 µg/mL chloramphenicol, 50 µg/mL ampicillin) and saturated cultures were kept at 4°C for a maximum of 1 week. Auto-induction was initiated by a 1:1,000 dilution of P-0.5G pre-culture in ZYP-5052 auto-induction medium (1% w/v casein tryptone, 0.5% w/v yeast extracts, 1 mM $MgSO_4$, 50 mM Na_2HPO_4, 50 mM KH_2PO_4, 25 mM $(NH_4)_2SO_4$, 0.5% w/v glycerol, 0.05% glucose, 0.2% α-lactose, 35 µg/mL chloramphenicol, 100 µg/mL ampicillin) and carried out for 48 h at 22°C and 300 rpm in a shaking incubator to generate non-classical inclusion bodies. Cells were harvested by centrifugation at 4,200 g for 15 minutes at 4°C, resuspended in lysis buffer (100 mM Tris pH 8.0, 1 mM EDTA) and lysed at 10,000-15,000 psi using an Emulsiflex-C5 homogenizer (Avestin Inc., Ottawa, ON, Canada). The lysates were centrifuged at 12,000 g for 30 min and pellets were quickly washed 3 times in 100 mM Tris pH 8.0. Resuspension and solubilisation of inclusion bodies was performed overnight in 50 mM Tris pH 8.0, 10 mM dithothreitol (DTT), 0.2% N-lauroyl sarcosine (5 mg of inclusion bodies per mL of solubilisation buffer). Solubilized inclusion bodies were concentrated to ~10 mg/mL protein concentration and folding was carried out as previously described[4]. Pepsin-activated CatK and V were conditioned with 2 M ammonium sulfate and purified by hydrophobic interaction chromatography (HIC) on an N-butyl Sepharose column using an AKTÄ system (GE Healthcare, Fairfield, Connecticut, USA). Eluted proteins were buffer-exchanged to eliminate remaining traces of ammonium sulfate (0.1 M sodium acetate, pH 5.5, 0.5 mM EDTA, 0.5

mM DTT) using an Amicon 10 kDa Ultra Concentrator (EMD Millipore) and loaded onto an SP-Sepharose column (GE Healthcare) as previously described[1]. Folded ProCatS was concentrated at 4°C with an Amicon 10 kDa Ultra Concentrator, then conditioned in 400 mM ammonium sulfate and purified once by HIC on a Phenyl Sepharose column as previously described [4]. The eluted pro-enzyme was buffer-exchanged (50 mM Tris pH 8.0, 2 mM EDTA) and autocatalytic maturation of the pro-enzyme was performed at 26°C for 5 hours at pH 4.6 and the reaction was stopped by dropwise addition of 1.5 M 2-(4-Morpholino)ethane Sulfonic Acid (MES) buffer pH 6.9 until pH reached 5.5.

Supplementary References

1. Linnevers, C. J. *et al.* Expression of human cathepsin K in Pichia pastoris and preliminary crystallographic studies of an inhibitor complex. *Protein Sci.* **6**, 919–921 (1997).
2. Brömme, D., Li, Z., Barnes, M. & Mehler, E. Human cathepsin V functional expression, tissue distribution, electrostatic surface potential, enzymatic characterization, and chromosomal localization. *Biochemistry* **38**, 2377–2385 (1999).
3. Studier, F. W. Protein production by auto-induction in high density shaking cultures. *Protein Expr. Purif.* **41**, 207–234 (2005).
4. Kramer, G. *et al.* Optimized folding and activation of recombinant procathepsin L and S produced in Escherichia coli. *Protein Expr. Purif.* **54**, 147–156 (2007).

Supplementary figure legends

Figure S1: Bovine neck elastin was either incubated alone or with active CatK, or E64 inactivated CatK prior to mineralization. Both Intact and digested mineralized elastin was decalcified with 0.6 N HCl and acid extracts were analyzed for calcium and phosphate content. Pairwise comparison of calcium/phosphate levels between control elastin (n = 5) and digested samples (n = 5 for each set) was carried out by Mann-Whitney U test (**: $p < 0.01$).

Figure S2: MOVAS-1 cells were incubated in low phosphate complete DMEM (1 mM Pi) in the presence or absence of BSA tryptic digest, of the synthetic peptide VGVAPG or CatK, S, V and MMP-12 elastin digestion products (10 µg/mL) for 21 days and cell layers were stained with 2% Alizarin Red pH 4.2. Images were recorded at 20x magnification. Thresholding with NIS-element software did not allow the detection of an Alizarin-Red positive signal.

Figure S3: MOVAS-1 cells were incubated in low phosphate (LP) complete DMEM (1 mM Pi) or high phosphate (HP) complete DMEM (2 mM Pi) supplemented with the ERK inhibitor FR180204 (1 µM) in the presence or absence of a tryptic BSA digest, synthetic

VGVAPG peptide or CatK-, S-, V or MMP-12-digested elastin (10 µg/mL) for 21 days. a) Cell layers were stained with 2% Alizarin-Red pH 4.2 (calcium staining), images were recorded at 20x magnification and b) the intensity of Alizarin-Red-positive staining was quantified with NIS-Elements software (Nikon), data are represented as mean ± SD. c) Cytochemical staining of active alkaline phosphatase was carried out with an alkaline phosphatase detection kit (Sigma-Aldrich) and d) staining intensity was quantified with NIS-Elements software (Nikon), data are represented as mean ± SD. e) Cell layers were decalcified with 0.6 N HCl for 24 hours and solubilized calcium was quantified by cresolphthalein assay (Randox) and normalized to protein concentration. Comparison between control HP (n = 4) and peptides treated HP samples (n = 4 for each set) was carried out with the one-way Mann-Whitney U test (n.s.: non-significant).

Figure S4: a) 4 mm long aortic rings were incubated in high phosphate (HP) αMEM (2 mM Pi) in the presence or absence of E64 and GM6001 (10 µM) or lactose (50 mM) for 12 days at 37°C. 5 µm sections were stained with Alizarin Red (calcium stain) or Hematoxylin Eosin and images were recorded at 1000 x magnification**.**

Figure S5: a) 4 mm long aortic rings were incubated in low phosphate (LP) complete αMEM (1 mM Pi) or in high phosphate (HP) αMEM (2 mM Pi) in the presence or absence of E64 and GM6001 (10 µM) or lactose (50 mM) for 12 days at 37°C. 5 µm sections were stained with Sirius-Red (collagen stain) and images were recorded at 100x magnification**.** b) Sirius-Red intensity was quantified with NIS-Elements software (Nikon), data are represented as mean ± SD**.**

Supplementary figure

Figure S1

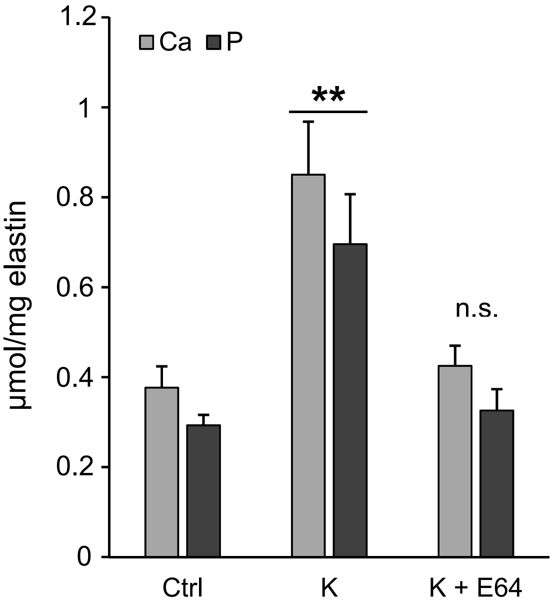

Figure S2

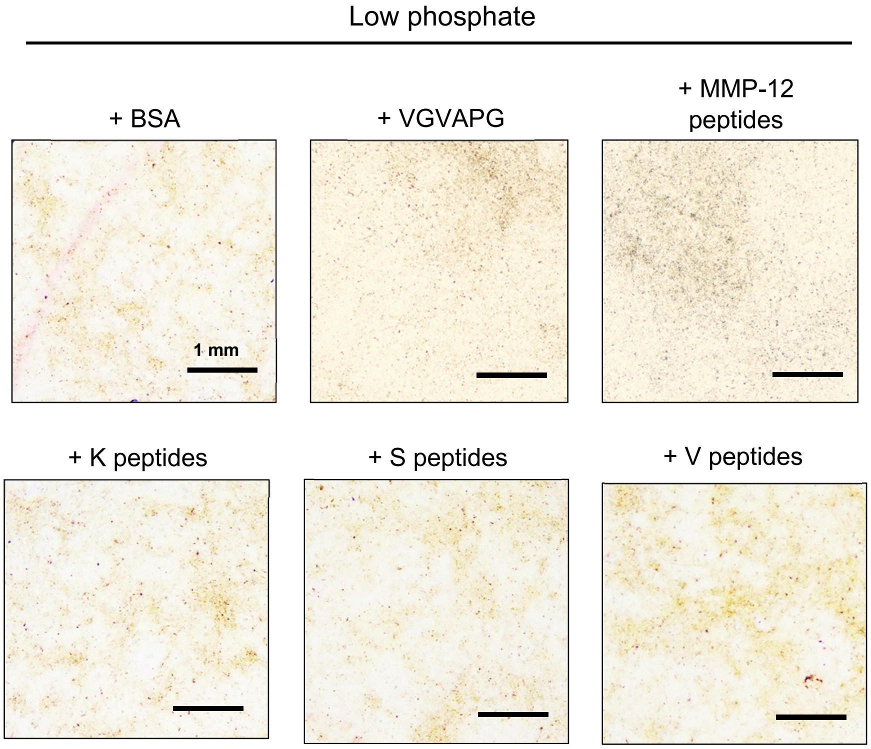
Low phosphate
+ BSA
+ VGVAPG
+ MMP-12 peptides
1 mm
+ K peptides
+ S peptides
+ V peptides

Figure S3

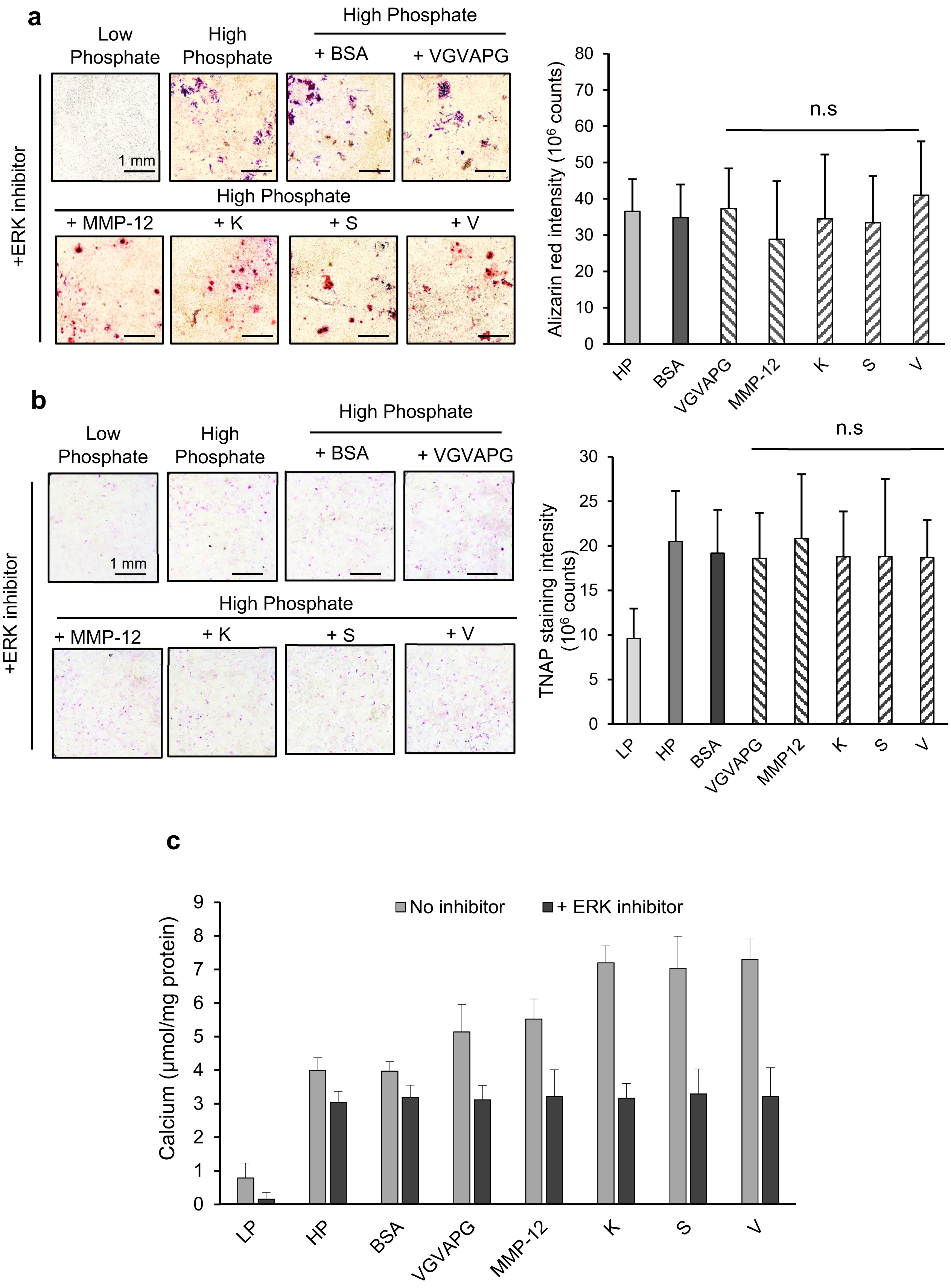
a
Low Phosphate
High Phosphate
High Phosphate
+ BSA
+ VGVAPG
+ERK inhibitor
1 mm
High Phosphate
+ MMP-12
+ K
+ S
+ V
Alizarin red intensity (10^6 counts)
n.s
HP
BSA
VGVAPG
MMP-12
K
S
V
b
Low Phosphate
High Phosphate
High Phosphate
+ BSA
+ VGVAPG
+ERK inhibitor
1 mm
High Phosphate
+ MMP-12
+ K
+ S
+ V
TNAP staining intensity (10^6 counts)
n.s
LP
HP
BSA
VGVAPG
MMP12
K
S
V
c
No inhibitor
+ ERK inhibitor
Calcium (µmol/mg protein)
LP
HP
BSA
VGVAPG
MMP-12
K
S
V

Figure S4

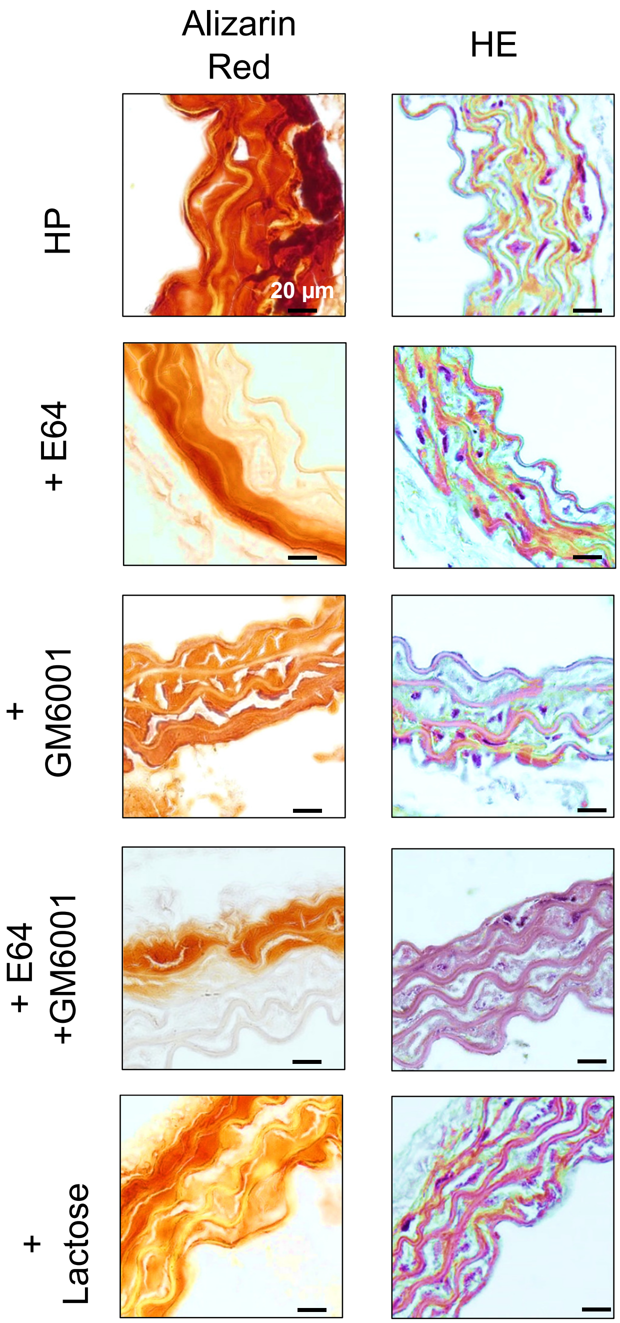

Figure S5

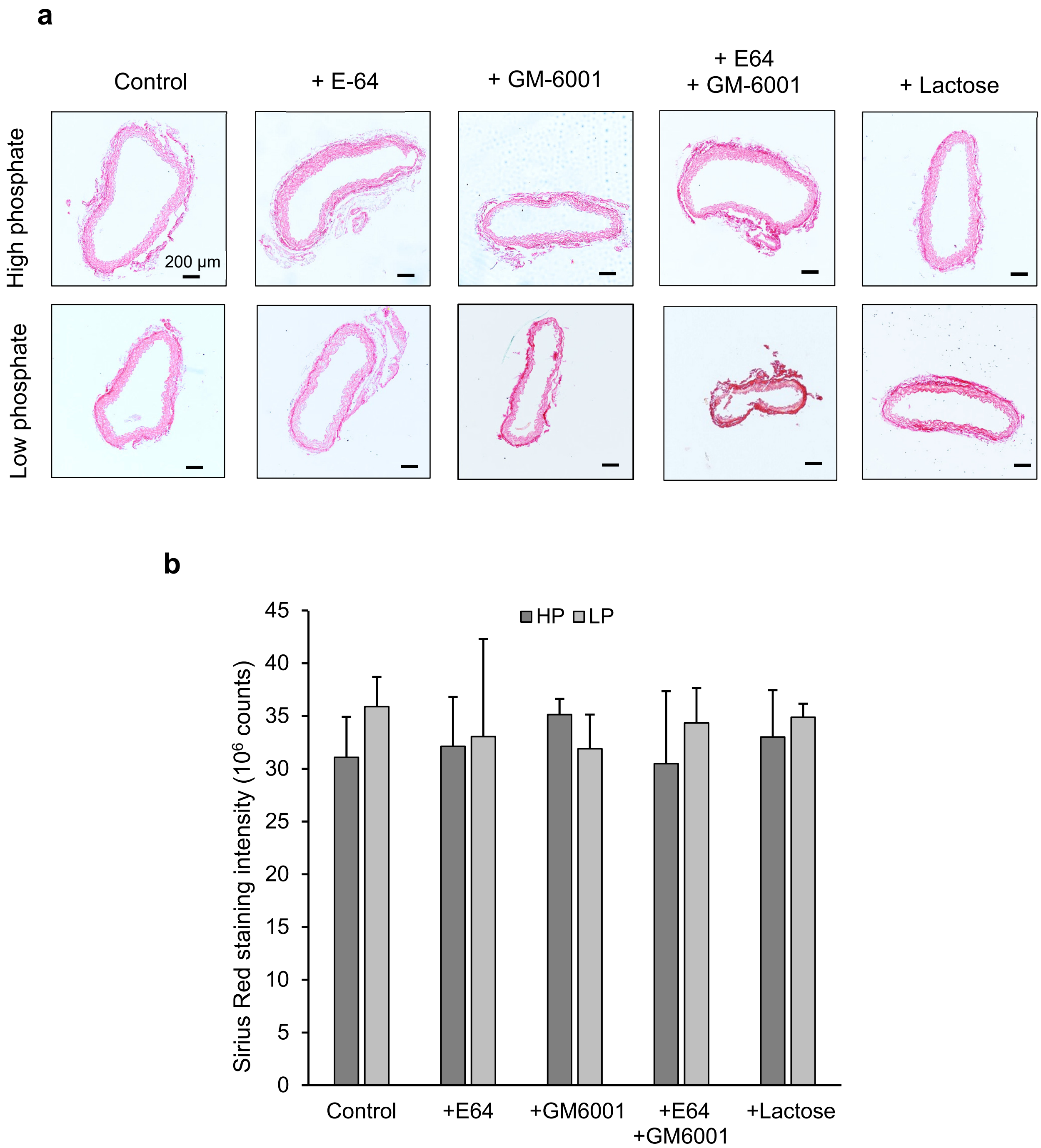
a
Control
+ E-64
+ GM-6001
+ E64
+ GM-6001
+ Lactose
High phosphate
Low phosphate
200 µm
b
HP
LP
Sirius Red staining intensity (10^6 counts)
45
40
35
30
25
20
15
10
5
0
Control
+E64
+GM6001
+E64
+GM6001
+Lactose

Non-cropped Western blot used in Figure 4

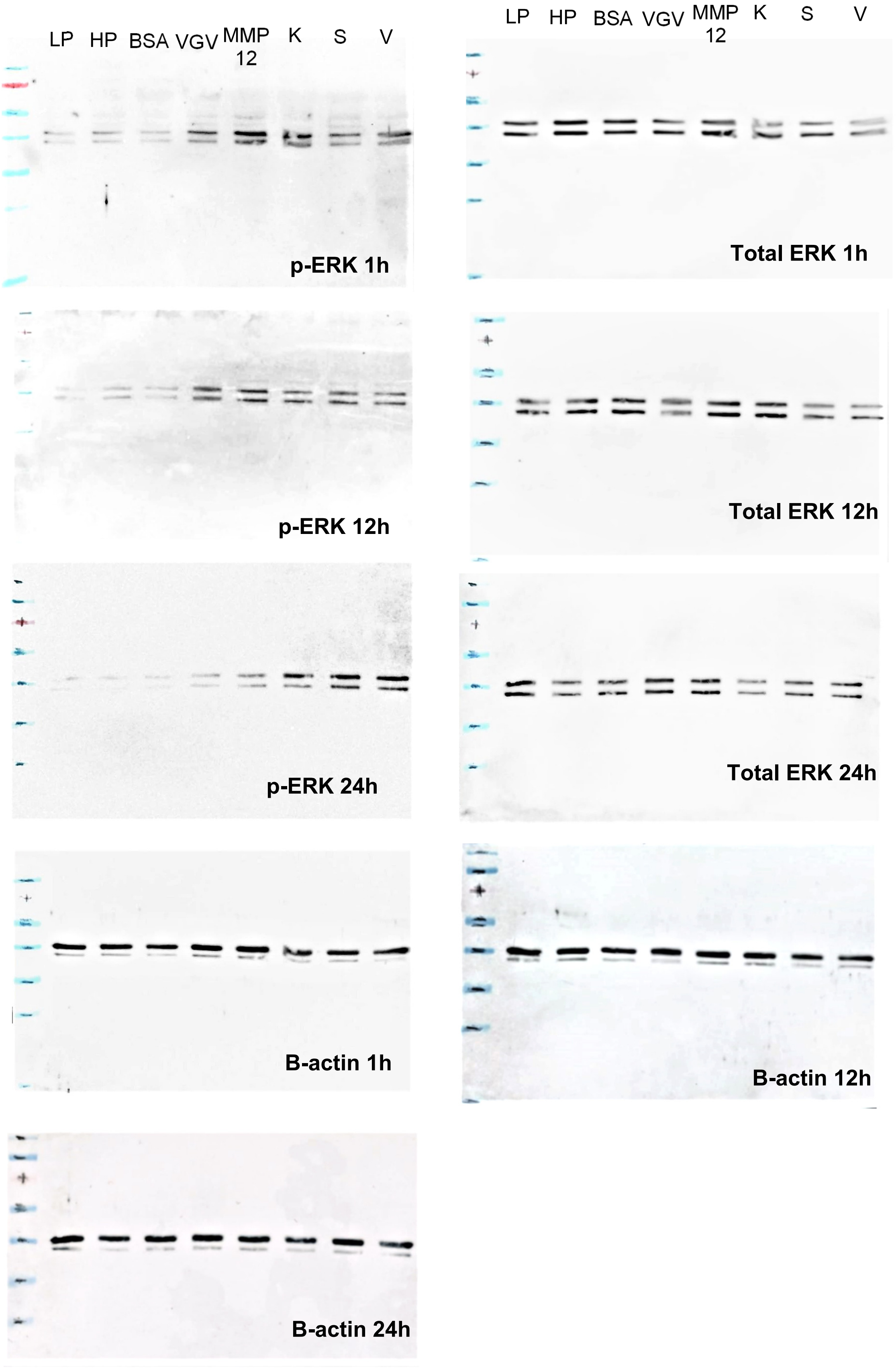

Supplement: Supplementary file 1 — Supplementary data [file 41598_2019_45918_MOESM1_ESM.pdf]
